# Human Rights and the ASEAN Smart Cities Network: Covering Unaddressed Civic and Social Concerns

**DOI:** 10.12688/f1000research.167098.2

**Published:** 2026-01-10

**Authors:** Bama Andika Putra

**Affiliations:** 1 School of Sociology, Politics and International Studies, University of Bristol, Bristol, England, UK; 2 Department of International Relations, Universitas Hasanuddin Fakultas Ilmu Sosial dan Ilmu Politik, Makassar, South Sulawesi, Indonesia

**Keywords:** Smart City, ASEAN2, ASEAN Smart Cities Network3, Human Rights4, Social Concerns5

## Abstract

The development of smart cities through the ASEAN Smart Cities Network (ASCN) has accelerated the growth of Southeast Asia’s capital and important cities. However, a growing concern has been how much the intentions of Southeast Asian cities to achieve ‘smart city status’ consider the basic elements of human rights and the provision of essential public services. The first policy recommendation is to acknowledge the vulnerability of the underprivileged, personal security, and social inclusion in governing smart cities to counter the possible derailment of democratic progress in the region. The second recommendation is to have fellow ASEAN member states assist in providing essential public services to avoid a ‘development’ model imposed by external funding stakeholders. The policy brief uses secondary data from 2018 to 2024 on the ASEAN Smart Cities Network projects and identifies civic and social concerns that arose during this period.

## Introduction: The challenges faced by Southeast Asia’s cities

The ASCN is the Association of Southeast Asian Nations (ASEAN) solution towards urbanization and city-based challenges. In 2018, Singapore led the initiative to establish the ASCN, believing that a ‘smart city’ conception of Southeast Asian states would help Southeast Asian nations address challenges arising from urbanization and the underdevelopment of larger ASEAN cities. ^1^ As Ludher argues, “Most of ASEAN’s growth has been and will continue to be driven by urban centers, with more people expected to urbanize by 2030”. ^2^ This policy brief perceives that throughout the process, the ASCN lacks proper consideration towards human rights and elements of sustainability (civic and social dimensions) in its plan to accelerate the growth of its member cities.

**Figure 1.**
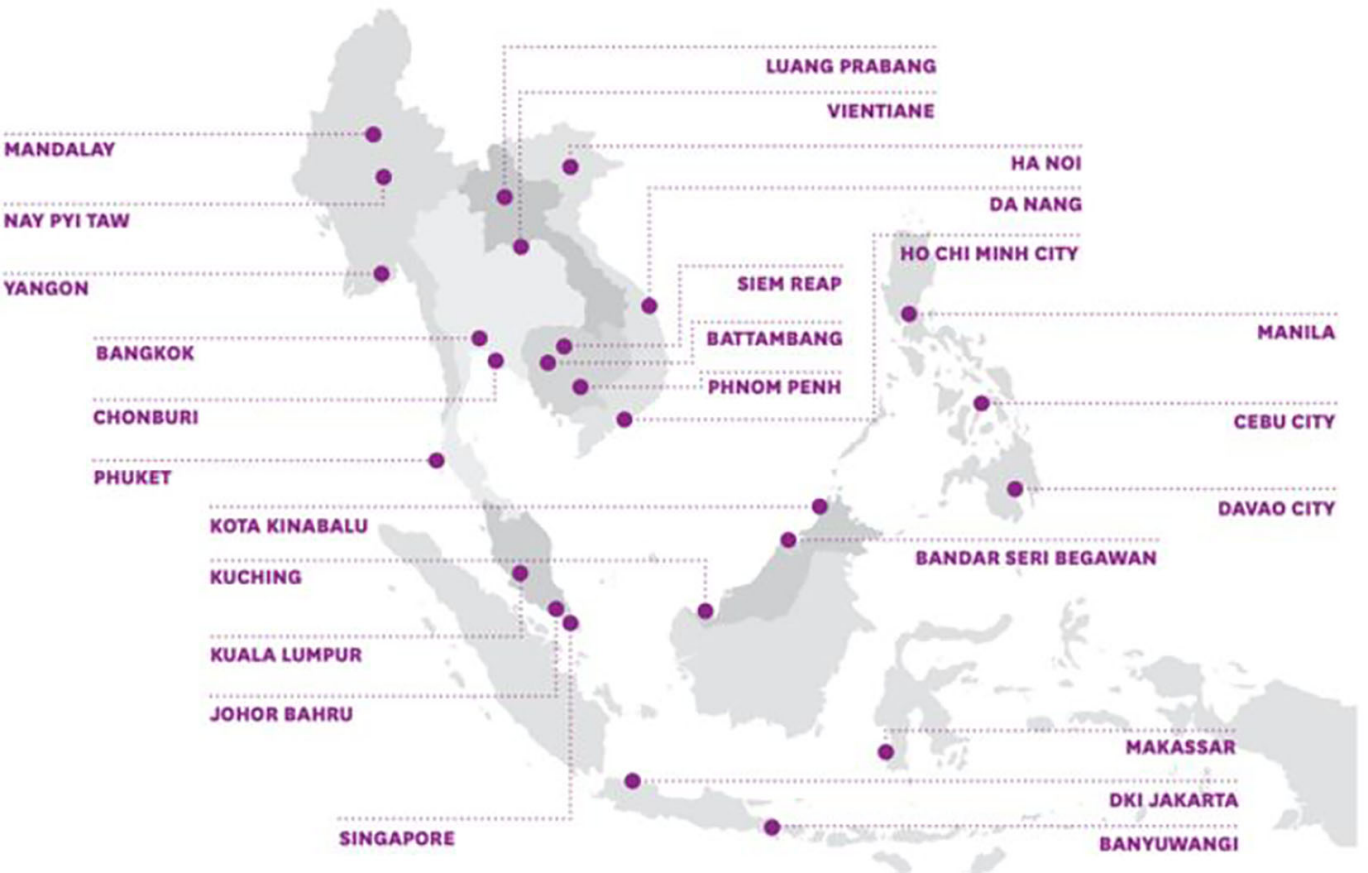
Member cities of the ASEAN Smart Cities Network. Source: Ludher (2018). ^2^

The ASCN started with 26 pilot cities across the ten ASEAN member states. As shown in Figure 1 below, these cities differ significantly in terms of population, political systems, and current levels of development. Nevertheless, the ASCN has emphasized that, through the network, normative guidelines will serve as the foundation for member cities’ smart city development. ^3, 4^ Perceiving the importance of city-level and scalable solutions, the ASCN was established at the 32nd ASEAN Summit 2018 and identified 26 Pilot cities, reflecting consistency with ASEAN’s regional institutionalism, which places heavy emphasis on stability and development. As of 2024, this list has increased to 31 cities, including Sihanoukville City, Sumedang, Rayong, Khon Kaen, and Chiang Mai.

The ASCN’s smart city conceptions aim to achieve a high quality of life, a competitive economy, and a sustainable environment. ^4^ ASCN would thus assist in establishing digital infrastructure, fostering partnerships, and providing funding for several of the ASCN’s key development areas. Among the key development areas this policy brief concerns are the ‘civic and social,’ comprising social cohesion, culture and heritage, tourism, public and municipal services, and governance. ^5^

In recent years, city groups have been working to address the issues cities collectively face in their development. Notable examples include Local Governments for Sustainability, the C40 Cities Climate Leadership Group, United Cities and Local Governments, WeGo, and the Strong Cities Network. These cities’ networks have been used to facilitate dialogue and the sharing of best practices concerning achieving sustainable smart cities. Several ASCN members are part of those city groupings, indicating that the sources of development practices they are informed about are diverse. Nevertheless, can the ASCN truly address the prevalent issues local governments in Southeast Asia face?

This policy brief identifies two issues arising from the ASCN, based on a normative and literature review of the initiative’s direction. They include the neglect of human rights and the imposition of a ‘developing’ model on financing stakeholders. The ASCN is geared to establish ‘smart cities’ in Southeast Asia. However, this region comprises states with significant disparities. The diverse social, economic, and political landscapes have already exacerbated human rights protection due to authoritarian and semi-authoritarian rule, which aim to maintain regime legitimacy by exerting greater power. Introducing information and communication technologies in the ASCN member cities risks increased surveillance and control in the region’s less democratic settings. As past studies have argued, smart cities do not directly correlate with sustainability, ^6, 7^ and the ASCN and the developed city master plans of the member cities are ambiguous about how the most disadvantaged would benefit from greater inclusivity in smart cities. ^8– 11^ Therefore, it could represent what a study mentioned as evolving social practices restricting rights. ^12^ This study acknowledges that there are cases of illiberal, yet effective, smart cities. However, given civic and social concerns, these practices should be carefully approached through the ASCN to ensure that developments in civic and social sustainability do not decline and to balance between liberal governance and the developmental state.

A second concern identified in this policy brief is the imposition of development models by external actors. The ASCN collaborates with and links ASCN members’ projects to potential financing from various stakeholders. The problem with this is that the ASCN eventually aims to achieve accelerated profit-driven growth, which includes broader privatization processes along the way. Observing the case of Phnom Penh’s forced privatized land that disadvantaged low-income people, ^11^ a “top-down” (terminology introduced by de Jong in 2023) approach thus risks the livelihoods of ASEAN citizens. ^8^

## Strengthening the ASEAN Smart Cities Network: Two Possible Solutions

This section argues that to complement the deficiencies of the ASCN’s smart city conception, two recommendations can be adopted. The first recommendation is to acknowledge the vulnerability of the underprivileged, provide personal security, and promote social inclusion for governing smart cities. As shown in Table 1 below, a prominent problem in Southeast Asian states is the lack of democracy in their social and political landscapes. Consequently, when the ASCN echoed the importance of smart cities, there were fears that this would further derail democratic progress in the region. Studies in the past have shown that smart-city developments risk more substantial violations of privacy rights, as high-tech companies have a stronger voice and role in the planning and financing of smart-city projects. ^13– 16^ Half of the Southeast Asian states are authoritarian or semi-authoritarian states. Consequently, there is an intense fear of abuse of power and increased surveillance capacities, which are feared to be the consequences of smart city conceptions, albeit the presence of institutional designs that help counter their intensity. ^7, 17^

**Table 1.** Southeast Asian states’ ranks in the 2023 Democracy Index.

Country	Global Rank	Regime Type
Malaysia	40	Flawed Democracy
Indonesia	56	Flawed Democracy
Thailand	63	Flawed Democracy
Singapore	69	Flawed Democracy
Philippines	53	Flawed Democracy
Cambodia	121	Authoritarian
Vietnam	136	Authoritarian
Laos	159	Authoritarian
Myanmar	166	Authoritarian
Brunei Darussalam	NA	NA

Source: Economist Intelligence Unit. ^18^

Given the vulnerability of the underprivileged in Southeast Asia’s larger cities, it is essential to examine how past studies have concluded that smart city developments can leave the poor and unemployed in isolation. ^19, 20^ This policy brief acknowledges that smart city development is geared toward economic progress despite the civic and social elements emphasized in the ASCN. The problem with this is the strong possibility that social inclusion and people’s participation in governance will be further hampered, as shown in the conclusions of past studies on this subject. ^21– 25^ Therefore, this destination toward ‘smart city’ status must be carefully pursued by the ASCN to ensure that, in the process, local governments do not impede upon the fundamental human rights of its citizens in the name of development.

This policy recommendation has both positive and negative implications. The positive implication is that the ASCN can argue its consistency with ASEAN’s recent approach to respecting human rights in the region. The diversity of the political landscape of Southeast Asia is seen with military coups, decades-long rule, lack of political participation, and rigged elections all over the region. ^26, 27^ Therefore, by acknowledging human rights elements in its smart city development plans, it is able to be consistent with the steps already taken to better human rights in the region. ^28– 30^ The efforts include the 2012 ASEAN Declaration on Human Rights and the ASEAN Intergovernmental Commission on Human Rights (AICHR). In recent years, ASEAN’s version of human rights and the AICHR have been protested for acknowledging human rights as dependent on regional and national contexts. However, these small steps are the most feasible for a region as diverse as Southeast Asia. Studies have shown how ‘smart city’ conceptions have been a dominant theme within states’ urban planning in recent years. ^31– 34^ Therefore, the best course of action is to complement what is lacking within the existing smart city conceptions of the ASCN.

Nevertheless, this recommendation also has negative implications. Among the most prominent is the possible violation of the ‘ASEAN Way.’ ASEAN was established on the importance of consensus-based decision-making, non-interference, non-intervention, and the peaceful resolution of conflicts. ^35, 36^ Despite the vast human rights concerns across the region, ASEAN has never acted as an intervening body and acknowledges that all Southeast Asian states are unique in their respect. Emphasizing the importance of specific human rights elements in the smart city development process could provoke rejection by ASEAN member states that are sensitive to human rights discourse, such as Lao PDR, Myanmar, Cambodia, Vietnam, and Brunei Darussalam. ^37, 38^

The second recommendation is for fellow ASEAN member states to assist in providing essential public services. Under its current model, the ASCN connects ASCN members with potential private financing or financing from ASEAN’s external partners. The 2022 UN Human Development Index (HDI) in Table 2 below shows a significant disparity among ASEAN members. The HDI is measured using several indicators, including health, education, and standard of living. ^39^ In Southeast Asia, states like Singapore ranked among the best globally, at 9th, and Cambodia and Myanmar ranked 148th and 144th. In understanding the disparity in the rankings, the stability of the government and its ability to deliver basic needs to the people (through public services) are essential elements. Therefore, this policy brief perceives that to achieve sustainable smart city status, ASEAN member states need to incorporate within the development model direct assistance provided by higher-ranked states in the HDI (such as Singapore, Brunei Darussalam, and Malaysia) to ensure that ASCN cities don’t just attempt to achieve the smart city status without proper consideration towards the people’s welfare inclusively.

**Table 2.** Southeast Asian states’ ranks in the 2022 Human Development Index.

Country	Global rank	Total score (/1)
Singapore	9	0.949
Brunei Darussalam	55	0.823
Malaysia	63	0.807
Thailand	66	0.803
Vietnam	107	0.713
Indonesia	112	0.710
Philippines	113	0.620
Lao PDR	139	0.608
Myanmar	144	0.600
Cambodia	148	0.726

Source: United National Human Development Index (2024). ^39^

The problem identified with the ASCN’s current model is the acceleration of development under the financing stakeholder’s model. In the past, many of ASEAN’s external partners have partnered with the ASCN pilot cities. ^40^ Japan has been an active nation, aiming to adopt Japanese models of sustainability and smart city infrastructure through its Japan International Cooperation Agency (JICA), which has increased its presence in Siem Reap, Phnom Penh, and Yangon City. ^10, 11^ China’s Belt and Road Initiative (BRI) has also financed many of Southeast Asia’s regional strategic infrastructures. ^41^ Some have been under the framework of the BRI, while others have been through partnerships with China’s private and public industries. Huawei, for example, was reported to contribute to Thailand’s digital economy and technological updates. ^8, 42^ The problem with this model is that the intention to construct this smart city leads Southeast Asian states to be influenced by the national interests of foreign states, which aim to impose their development model on ASCN members. ^43, 44^ Consequently, it will be difficult for recipient cities of the financing to cater to the needs of their vulnerable groups, as attention would be diverted to accelerating development in line with the interests of the financing stakeholders. Nevertheless, this problem is somewhat countered by the ASEAN member states’ capacity negotiate conditions, which could partially counter these concerns.

The proposed model, therefore, generates a possible favorable implication for the ASEAN organization. By assisting fellow members, this policy aligns with ASEAN’s approach to addressing development gaps among its members. In the past, when trade agreements between ASEAN and external partners were perceived as distortive for a member’s economy, the standard solution has been to provide distinct solutions for the less developed nations of ASEAN, known as the CMLV (Cambodia, Myanmar, Laos, Vietnam). ^45– 47^ Rather than forcing those nations to adopt free trade measures immediately, unique treatments are provided through extended deadlines and the exclusion of commodities deemed sensitive to the state.

Nevertheless, assistance from fellow ASEAN members also raises concerns. Singapore is the only nation that has developed enough in human development categories to provide such assistance. Even with the following ranked nations (Brunei, Malaysia, and Thailand), the systems have not been the most stable and consistent in delivering essential basic public services to their people. In Thailand, for example, the country has been a victim of undemocratic rule and coups over the past two decades. ^48, 49^ Therefore, the question is whether Singapore would be willing to openly assist all of its ASEAN counterparts in establishing a balance between smart city status and sustainable development.

## Actionable recommendation: Moderate adoptions

Based on two policy recommendations in the previous section, the actionable recommendation is a moderate application of both policies. In the ASEAN context, it is crucial to consider its member states’ sensitivity to any notions that may impede the ASEAN Way. Therefore, in the first recommendation that acknowledges the vulnerability of the underprivileged, the terms used must ensure that they refrain from the language of ‘enforcing’ or ‘must.’ An example of alternative terms that can be used is ‘encourage’ or ‘strongly consider,’ which avoids any notion of forcing ASEAN member states, represented by the ASCN members, to abide by human rights principles in their efforts to achieve smart city status. Taking, for example, the AICHR, despite the commission’s mandate to engage in the protection and promotion of human rights in Southeast Asia, it is observed that the AICHR has focused more on promotional mandates than on protection, to ensure continued support from all ten member states. ^50, 51^

With the second recommendation to have fellow ASEAN member states assist in providing essential public services, the moderate adoption of this policy is to identify key areas of public services in which a member state can assist. Rather than choosing Singapore, for example, to take on the burden of helping ASEAN members alone, the ideal mechanism is to assign the responsibility for a particular dimension of public service assistance to one member state and another field to another. For example, Malaysia can assist ASCN members in exploring possible mechanisms to enhance the health of its citizens. Meanwhile, Singapore can be entrusted with overall city planning to ensure balanced human development across health, education, and the standard of living.

## Conclusion

This policy brief examines several recent trends arising from ASEAN’s ASCN, aiming to establish smart city status among ASCN members. Smart city status does not automatically lead to sustainability. Therefore, additional measures need to be taken by the ASCN members to ensure that it enhances its respect for civil and social needs, which may be neglected throughout the improved development process. Based on secondary data from 2018 to 2024, this study concludes that two recommendations can be adopted to promote more sustainable city-based development across the Southeast Asian region.

The recommendations include acknowledging the vulnerability of the underprivileged, ensuring personal security, promoting social inclusion in governing smart cities, and having fellow ASEAN member states assist in providing essential public services. These recommendations address several disparities and diversities among ASEAN member states, as reflected in the 2024 Democracy Index and the 2022 Human Development Index. Furthermore, it considers several problems arising from existing ASCN models for developing cities, including the risk of violating privacy rights, a lack of social inclusivity in city development, and the imposition of development models by external funders. In conclusion, there is no clear linkage between the ASCN and sustainable measures while accelerating its member cities’ growth. Thus, this policy brief recommends that the ASCN take a step back and consider what is deficient within its development models to adopt more inclusive policies in its future development.

## Ethical considerations

Ethical approval and consent were not required.

## Data Availability

The dataset used in this study is publicly available and sourced from reputable organizations. All data can be accessed through their official platforms, with the detailed data and links accessible below:
• UN Development Index 2022: https://hdr.undp.org/data-center/human-development-index#/indicies/HDI; • Democracy Index 2023: https://www.eiu.com/n/campaigns/democracy-index-2023/. All data required to replicate the findings of this study are available on those websites, on which users can filter based on the inquired variable and period.
