# Supplementary material for: Human Rights and the ASEAN Smart Cities Network: Covering Unaddressed Civic and Social Concerns
Source: F1000Res. 2026 Mar 11;14:733. Originally published 2025 Jul 25. [Version 3] doi: 10.12688/f1000research.167098.3 (PMC13032098; doi:10.12688/f1000research.167098.3)
Supplement: Peer review history

# Reviewer report: Reviewer response for version 3

**DOI:** 10.5256/f1000research.197288.r467244

**Published:** 2026-03-27

**Authors:** Renju Chandran, Sarath Chandran MC

**Affiliations:** 1 Chinmaya Vishwavidyapeeth (Deemed to be University), Ernakulam, Kerala, India; 2 Amrita School of Arts, Humanities and Commerce, Amrita Vishwa Vidyapeetham - Amritapuri Campus (Ringgold ID: 301814), Amrithapuri, Kerala, India

The paper provides a timely and policy-relevant discussion on the intersection of smart cities, governance, and human rights in ASEAN. It makes a valuable contribution by shifting attention from technocratic smart city narratives to civic and social sustainability concerns. However, the manuscript currently reads more as a normative policy essay rather than a rigorous academic policy analysis, and requires major revisions to enhance methodological depth, analytical balance, and scholarly contribution.

- The argument appears overly one-sided, as it assumes a direct relationship between smart city development and human rights risks without adequately considering potential positive outcomes such as improved governance, efficiency, and service delivery.
- The study relies entirely on secondary data, and the absence of case studies, primary evidence, or city-level analysis limits the robustness and practical relevance of the conclusions.
- The concept of “human rights” is not clearly defined, and the paper does not distinguish between civil–political rights and socio-economic rights, which is particularly important in the ASEAN context.
- The use of the Democracy Index and Human Development Index is descriptive, but the manuscript does not establish a clear analytical link between these indicators and the effectiveness of the ASEAN Smart Cities Network.
- The discussion treats the ASEAN Smart Cities Network as a homogeneous structure, without adequately accounting for variations across cities and the role of local governments in shaping smart city initiatives.
- The central argument presents a linear relationship between technology, surveillance, and democratic decline, but does not sufficiently consider the role of institutional safeguards or governance mechanisms that may mitigate such risks.
- The policy recommendations, while conceptually relevant, lack operational clarity, particularly regarding implementation mechanisms, responsible actors, and monitoring frameworks.
- The manuscript would benefit from stronger theoretical positioning, particularly by engaging with debates on developmental state models, institutional governance, and technology–society interactions.

Does the paper provide a comprehensive overview of the policy and the context of its implementation in a way which is accessible to a general reader?

Partly

Is the discussion on the implications clearly and accurately presented and does it cite the current literature?

Partly

Are the recommendations made clear, balanced, and justified on the basis of the presented arguments?

Partly

Reviewer Expertise:

Sustainable livelihood, Sustainability, Green Finance, Smart Cities.

We confirm that we have read this submission and believe that we have an appropriate level of expertise to confirm that it is of an acceptable scientific standard, however we have significant reservations, as outlined above.

## References

[1] : Examining the role of smart cities and sustainability research in achieving SDGs through bibliometric lens. *Discover Sustainability* .2025;6(1) : 10.1007/s43621-025-01596-w 10.1007/s43621-025-01596-w

[2] : Green finance and sustainability: mapping research development through bibliometric analysis. *Discover Sustainability* .2024;5(1) : 10.1007/s43621-024-00549-z 10.1007/s43621-024-00549-z

# Reviewer report: Reviewer response for version 3

**DOI:** 10.5256/f1000research.197288.r466652

**Published:** 2026-03-13

**Authors:** Tooran Alizadeh

**Affiliations:** 1 The University of Sydney, Sydney, New South Wales, Australia

Thanks for taking up my earlier review comments.

Does the paper provide a comprehensive overview of the policy and the context of its implementation in a way which is accessible to a general reader?

Yes

Is the discussion on the implications clearly and accurately presented and does it cite the current literature?

Partly

Are the recommendations made clear, balanced, and justified on the basis of the presented arguments?

Partly

Reviewer Expertise:

Urbanism, Southern Urbanism, Smart Urbanism, Digital Geographies, Infrastructure

I confirm that I have read this submission and believe that I have an appropriate level of expertise to confirm that it is of an acceptable scientific standard.

# Reviewer report: Reviewer response for version 2

**DOI:** 10.5256/f1000research.194955.r461534

**Published:** 2026-02-27

**Authors:** Tooran Alizadeh

**Affiliations:** 1 The University of Sydney, Sydney, New South Wales, Australia

This policy brief raises an important and timely intervention into the development trajectory of the ASEAN Smart Cities Network (ASCN), particularly by foregrounding human rights and social inclusion concerns that are often marginalised in technocratic smart city discourses. Its claim - that ASCN initiatives risk neglecting civic and social sustainability by reinforcing authoritarian governance and surveillance capacities while privileging growth-oriented, infrastructure-driven development - is both normatively compelling and regionally relevant.

 The brief is persuasive in identifying two structural tensions:

1. The potential marginalisation of vulnerable communities in technocratic smart city planning; and
2. The influence of external financing actors in shaping development trajectories.

However, while the recommendations are normatively sound, the second proposal - encouraging stronger ASEAN member states to assist others in delivering essential public services - raises feasibility concerns. ASEAN’s institutional design, shaped by the 'ASEAN Way' of non-interference and consensus, limits redistributive or interventionist mechanisms. Moreover, the political willingness of higher-ranked states to assume sustained developmental responsibility for others remains uncertain. As such, this recommendation, while principled, may face significant structural constraints.

 What the brief could strengthen is its engagement with bottom-up and grassroots smart initiatives. Globally, civic society actors have increasingly used digital tools to advance human rights, participatory governance, and community-based service provision - from the margins (Alizadeh & Prasad, 2025a, 2025b; Vadiati, 2022). These collective efforts demonstrate that ‘smart’ need not be synonymous with top-down, state-led, or corporate-driven infrastructure projects. In many contexts, civic tech movements, open-data communities, and local innovation labs have leveraged technology to enhance transparency, inclusion, and accountability (Nancy Odendaal, 2019; N. Odendaal, 2021).

 Incorporating this dimension could lead to a more actionable and politically feasible recommendation:

- Encourage ASCN members to identify, support, and scale bottom-up smart initiatives already operating within their cities;
- Create mechanisms within the ASCN framework to connect grassroots initiatives across member states for peer learning and knowledge exchange;
- Promote small-scale funding or recognition schemes that incentivise civic innovation aligned with human rights and social inclusion goals.

Such a recommendation would not directly challenge the ASEAN Way, nor would it depend on heavy redistributive commitments among member states. Instead, it would expand the definition of ‘smart city’ beyond infrastructure and investment, embedding civic participation within the ASCN’s mandate. Compared to the proposal of inter-state assistance in public service provision, this approach may be more politically viable and institutionally compatible with ASEAN’s consensus-driven structure.

 Overall, the policy brief makes a valuable intervention by insisting that smart city development must be evaluated through a human rights lens. Strengthening the recommendations to include support for grassroots smart initiatives would further enhance its practical relevance and align its normative commitments with emerging global urban governance practices.

Does the paper provide a comprehensive overview of the policy and the context of its implementation in a way which is accessible to a general reader?

Yes

Is the discussion on the implications clearly and accurately presented and does it cite the current literature?

Partly

Are the recommendations made clear, balanced, and justified on the basis of the presented arguments?

Partly

Reviewer Expertise:

Urbanism, Southern Urbanism, Smart Urbanism, Digital Geographies, Infrastructure

I confirm that I have read this submission and believe that I have an appropriate level of expertise to confirm that it is of an acceptable scientific standard, however I have significant reservations, as outlined above.

## References

[1] : Alternative smart urbanism: An intersectional feminist take on learning from the margins. *Digital Geography and Society* .2025;9: 10.1016/j.diggeo.2025.100140 10.1016/j.diggeo.2025.100140

[2] : Women in, at the margins, and against the smart cities: lessons from India. *Social & Cultural Geography* .2026;27(2) : 10.1080/14649365.2025.2552194 215-233 10.1080/14649365.2025.2552194

[3] : Everyday urbanisms and the importance of place: Exploring the elements of the emancipatory smart city. *Urban Studies* .2021;58(3) : 10.1177/0042098020970970 639-654 10.1177/0042098020970970

[4] : Alternatives to smart cities: A call for consideration of grassroots digital urbanism. *Digital Geography and Society* .2022;3: 10.1016/j.diggeo.2022.100030 10.1016/j.diggeo.2022.100030

# Response

**Published:** 2026-03-02

**Authors:** bama andika putra

**Affiliations:** University of Bristol School of Sociology Politics and International Studies, Bristol, England, UK

Thank you for the comments. After reviewing the second recommendation, I have decided to adopt your proposed recommendation to utilize bottom-up initiatives to replace the policy of ASEAN member states being pushed to assist one another. It acknowledges that the previous second recommendation would not be politically feasible to adopt, due to the significant structural constraints. Therefore, as elaborated in the second recommendation (with slight changes to the wording in the abstract and conclusion sections), the revised second recommendation acknowledges that civic society has increasingly used digital tools to accelerate participatory governance and human rights. Therefore, by acknowledging the significance of existing bottom-up initiatives, this recommendation would underscore the importance of the ASCN scaling bottom-up smart initiatives already operating, enhancing information and knowledge exchange among member states, and promoting small-scale funding. Nevertheless, it still incorporates the first revision’s second recommendation’s concerns on the disparity among ASEAN member states across several public service dimensions, which is now used as a potential negative consequence of the revised second proposal.

# Reviewer report: Reviewer response for version 1

**DOI:** 10.5256/f1000research.184179.r434955

**Published:** 2026-01-02

**Authors:** Tran Xuan Hiep

**Affiliations:** 1 The University of Danang, University of Science and Education, Da Nang, Vietnam

1. Strengths of the Paper

**First**, the choice of topic has high academic and practical value. The paper approaches ASCN from a human rights perspective – a dimension that has been relatively underemphasized in smart city studies in ASEAN. The linkage between technology – surveillance – political institutions is appropriate to the Southeast Asian context.

**Second**, the arguments are theoretically grounded and supported with rich evidence. The author draws on many reputable academic sources (Democracy Index, HDI, studies on smart cities and surveillance capitalism), showing an effort to situate ASCN within the broader picture of global governance and sustainable development.

**Third**, the policy approach is pragmatic. The recommendation of “moderate adoption” reflects an understanding of ASEAN’s particularities, avoiding an idealized approach or rigid imposition of Western norms.

 2. Limitations and Points for Debate

 2.1. Evaluation of Theoretical Framework and Approach

**2.1.1. Normative approach dominates, lacking balance with empirical analysis** The paper mainly assesses ASCN from the perspective of human rights and liberal democracy, treating these as central standards for judging the success or failure of the smart city model. While this critical approach has value, it also creates a clear normative bias.

 The author almost assumes by default that:

- increased technology adoption + semi-authoritarian/authoritarian context = human rights violations.

However, the paper does not:

- compare with cases of “illiberal but effective smart cities” (e.g., Singapore, some Chinese cities);
- consider the perspective of ASEAN’s regional institutionalism, where stability and development are often prioritized over rapid democratization.

**Academic limitation:** The paper has not situated ASCN within the broader debate between *liberal governance* and the *developmental state*, which is highly characteristic of Southeast Asia.

**2.1.2. Lack of clarity on the concept of “human rights” in the ASEAN context** The author uses human rights as an almost universal category, without distinguishing between:

- civil–political rights
- economic–social–cultural rights

In ASEAN, many governments view access to public services, infrastructure, and employment as forms of human rights implementation, sometimes more important than political rights.

 The lack of differentiation makes the argument overly generalized, blurring the line between:

- actual human rights violations
- and differences in development priorities.

2.2. Critique of Research Methodology

**2.2.1. Complete reliance on secondary data** The paper uses secondary data from 2018–2024, but:

- no policy interviews,
- no urban citizen surveys,
- no specific case studies of ASCN cities.

This results in:

- assessments of surveillance and privacy violations that are structurally speculative;
- lack of direct evidence from specific ASCN projects.

This is a serious limitation for a policy brief, which requires practical evidence to support recommendations.

**2.2.2. Mechanical comparison of Democracy Index and HDI** Placing Democracy Index and HDI side by side illustrates ASEAN’s diversity, but:

- the paper does not prove a direct relationship between these rankings and ASCN’s effectiveness;
- it does not analyze changes over time (before–after joining ASCN).

The indices are used more as illustrations of viewpoints rather than causal analytical tools.

 2.3. Critique of Central Argument

**2.3.1. Linear argument “smart city → surveillance → democratic decline”** The author constructs a linear chain of reasoning: Technology → surveillance → control → rights violations.

 However, this approach:

- ignores the role of institutional design;
- does not analyze the potential for technology to be used for transparency, good governance, or improved service access.

This makes the paper lean more toward cautionary discourse than multidimensional analysis.

**2.3.2. Incomplete assessment of local governments’ active role** ASCN is described as a top-down imposed network, but in reality:

- many ASCN projects are proposed by city governments;
- the level of technology adoption varies greatly across cities.

Treating ASCN as a homogeneous structure reduces the accuracy of the argument.

**2.3.3. Critique of China and Japan’s role is overly politicized** The paper warns of major powers (BRI, JICA, Huawei) imposing development models. However:

- it does not compare loan conditions or policy constraints among partners;
- it does not analyze ASEAN countries’ capacity to negotiate and choose strategies.

This makes the argument appear more like geopolitical suspicion than evidence-based development assessment.

 2.4. Critique of Two Policy Recommendations

**2.4.1. Human rights recommendation: well-intentioned but impractical** Calling for human rights integration into smart city governance is reasonable. However:

- the paper does not specify concrete institutional mechanisms;
- it does not clarify who monitors, who implements, and who is accountable.

In ASEAN – where no enforcement mechanism exists – this recommendation is more symbolic than substantive.

**2.4.2. ASEAN intra-regional support recommendation: idealized view of Singapore’s role** The author suggests Singapore and high-HDI countries support others. However:

- ASEAN is not the EU, it has no common budget;
- Singapore has no legal obligation to support other countries;
- national interests may conflict with the role of “regional donor.”

This recommendation lacks assessment of political and economic incentives.

**Request: Major Revisions**

Does the paper provide a comprehensive overview of the policy and the context of its implementation in a way which is accessible to a general reader?

No

Is the discussion on the implications clearly and accurately presented and does it cite the current literature?

No

Are the recommendations made clear, balanced, and justified on the basis of the presented arguments?

No

Reviewer Expertise:

Political Sciences, International Relations, History

I confirm that I have read this submission and believe that I have an appropriate level of expertise to state that I do not consider it to be of an acceptable scientific standard, for reasons outlined above.

# Response

**Published:** 2026-01-07

**Authors:** bama andika putra

**Affiliations:** University of Bristol School of Sociology Politics and International Studies, Bristol, England, UK

**Reviewer 2, comment 2.1.1** : Normative approach dominates, lacking balance with empirical analysis;

 the paper does not: compare with cases of “illiberal but effective smart cities” (e.g., Singapore, some Chinese cities), consider the perspective of ASEAN’s regional institutionalism, where stability and development are often prioritized over rapid democratization. Academic limitation: The paper has not situated ASCN within the broader debate between *liberal governance* and the *developmental state*, which is highly characteristic of Southeast Asia.

**Response**: In the revised article, I aim to acknowledge the broader debate between liberal governance and the developmental state by directly acknowledging that the ASCN can pose a threat towards a balanced approach taken by these sub-unit variables. In Paragraph 2, Section 1, the revisions focused to acknowledge that the ASCN is a manifestation of ASEAN’s approach of prioritizing stability and development within its policies. The revised paragraph: “The ASCN started with 26 pilot cities across the ten ASEAN member states. As seen in Figure 1 below, these cities significantly differ based on the population, political systems, and current state of development. Nevertheless, the ASCN has emphasized that through the network, normative guidelines will act as the foundation for the member cities’ smart city development. ^3,^ ^4^ Perceiving the importance of city-level and scalable solutions, the ASCN was established at the 32 ^nd^ ASEAN Summit 2018 and determined 26 Pilot cities, which reflects consistency towards ASEAN’s regional institutionalism that places a heavy emphasis on stability and development. As of 2024, this list has increased to 31 cities, including Sihanoukville City, Sumedang, Rayong, Khon Kaen, and Chiang Mai.” Meanwhile, in the another paragraph (Paragraph 5, section 1), the revisions focused to acknowledge two things: 1) acknowledge that there are cases of illiberal, yet effective smart cities, which should open the case that there are other issues that can be considered within the case presented in this policy brief; 2) to balance between liberal governance and developmental state, ASCN in this case is criticized to potentially declining civic and social concerns. The revised paragraph: “This policy brief identifies two issues that arise from the ASCN. They include the negligence of human rights and imposing a ‘developing’ model from financing stakeholders. The ASCN is geared to establish ‘smart cities’ in Southeast Asia. However, this is a region consisting of states with significant disparities. The diverse social, economic, and political landscapes have already exacerbated human rights protection due to authoritarian and semi-authoritarian rules aiming to maintain their regime legitimacy through greater exertion of power. Introducing information and communication technologies in the ASCN member cities risks increased surveillance and control in the region’s less democratic settings. As past studies have argued, smart cities do not correlate directly to sustainability, ^6^^, ^^7^ and the ASCN and the developed city master plans of the member cities are ambiguous in showing how the most disadvantaged would benefit through greater inclusivity from smart cities. ^8–^ ^11^ Therefore, it could represent what a study mentioned as evolving social practices restricting rights. ^12 ^ This study acknowledges that there are cases of illiberal, yet effective smart cities. However, looking at civic and social concerns, these practices should be carefully approached through the ASCN to ensure that developments in civic and social sustainability does not decline, and can balance between liberal governance and the developmental state.”

**Reviewer 2, comment 2.1.2** : Lack of clarity on the concept of “human rights” in the ASEAN context; The author uses human rights as an almost universal category, without distinguishing between: civil–political rights & economic–social–cultural rights; In ASEAN, many governments view access to public services, infrastructure, and employment as forms of human rights implementation, sometimes more important than political rights; The lack of differentiation makes the argument overly generalized.

**Response**: In paragraph 5, section 1, I explain that the human rights concern here relates to the exertion of power by authoritiarian/ semi-authoritarian stakeholders in the Southeast Asian region. Fully acknowledging that the human rights conception differs between ASEAN and the other parts of the globe, I make it clear what the human rights concerns are for this policy brief: “Introducing information and communication technologies in the ASCN member cities risks increased surveillance and control in the region’s less democratic settings.” Furthermore, it is outsides the scope of this study to delve deep on the differences between ASEAN and the universal conception of human rights from a civil and economic perspective, as this opens up a whole different discussion on the suitability of ASEAN’s defined human rights conceptions to that of the West.

**Reviewer 2, Comment 2.2.1** . Complete reliance on secondary data; The paper uses secondary data from 2018–2024, but: no policy interviews, no urban citizen surveys, no specific case studies of ASCN cities. This results in: assessments of surveillance and privacy violations that are structurally speculative; lack of direct evidence from specific ASCN projects. This is a serious limitation for a policy brief, which requires practical evidence to support recommendations.

**Response**: I disagree with this suggestion. First, there is no issue with relying on secondary data, whether it be original research articles or policy briefs. In the context of policy brief, the angle taken for this study is one that argues the points based on normative and literature assessment angels, which does help policymakers look at the issue from diverse angles. In the past, this method is also used in multiple published policy briefs (see: https://research-information.bris.ac.uk/en/publications/aseans-climate-change-mitigation-and-adaptation-measures-abandoni/; https://research-information.bris.ac.uk/en/publications/the-politics-of-environmental-policy-haze-pollution-asean-and-the/). To emphasize this, I have revised the first sentence of Paragraph 5, section 1: “This policy brief identifies two issues that arise from the ASCN, based on a normative and literature assessment of the direction of the initiative.” Secondly, I disagree that as a result of this approach, the arguments are speculative. The first example of this potential issue has been raised in the Paragraph 6, section 1 taking in the case of Cambodia’s Phnom Penh: “Observing the case of Phnom Penh’s forced privatized lands that disadvantaged people with low incomes, ^11^ a “top-down” approach thus risks the livelihood of ASEAN citizens ^8^ ”. In addition to this, the specific cases that uses the study case of ASCN is on the second recommendation, that recommends ASEAN member states to provide essential public services. In Paragraph 6, Section 2, it is argued the cities that reflect this: “The problem identified with the ASCN’s current model is the acceleration of development under the financing stakeholder’s model. In the past, many of ASEAN’s external partners have partnered with the ASCN pilot cities. ^40 ^Japan has been an active nation, aiming to adopt Japanese models of sustainability and smart city infrastructure through its Japan International Cooperation Agency (JICA), which has increased its presence in Siem Reap, Phnom Penh, and Yangon City. ^10,^ ^11^ China’s Belt Road Initiative (BRI) has also financed many of Southeast Asia’s regional strategic infrastructures. ^41 ^Some have been under the framework of the BRI, while others have been through partnerships with China’s private and public industries. Huawei, for example, was reported to contribute to Thailand’s digital economy and technological updates. ^8,^ ^42^ The problem with this model is that the intention of constructing this smart city leads Southeast Asian states to be affected by the national interests of the foreign states, aiming to impose their development model on the ASCN members. ^43,^ ^44^ Consequently, it will be difficult for recipient cities of the financing to cater to the needs of their vulnerable groups, as attention would be diverted to accelerating the progress of the development in alignment with the interests of the financing stakeholder.”

**Reviewer 2, comment 2.2.2:** Mechanical comparison of Democracy Index and HDI Placing Democracy Index and HDI side by side illustrates ASEAN’s diversity, but: the paper does not prove a direct relationship between these rankings and ASCN’s effectiveness; it does not analyze changes over time (before–after joining ASCN). The indices are used more as illustrations of viewpoints rather than causal analytical tools.

**Response**: The two tables used (Democracy Index and HDI) are utilized to support the arguments made that act as background context in understand the specifics of the recommendations. Table 1 focuses on Southeast Asia’s Democracy Index that solidifies the claim of the lack of democracy within the Southeast Asian state’s social and political landscapes. Table 2 shows the disparity of human development among Southeast Asian states. The keyword here is ‘DISPARITY,’ allowing readers to understand the unique challenges of the Southeast Asian region, and steer clear from making the assumption that all Southeast Asian states are the same.

**Reviewer 2, comment 2.3.1.** Linear argument “smart city → surveillance → democratic decline” The author constructs a linear chain of reasoning: Technology → surveillance → control → rights violations. However, this approach: ignores the role of institutional design;, does not analyze the potential for technology to be used for transparency, good governance, or improved service access. This makes the paper lean more toward cautionary discourse than multidimensional analysis.

**Response**: In the revised paragraph 1, section 2, I make it clear that there is the presence of institutional designs that have the capacity to counter the significance of the issue. Nevertheless, it is also important to acknowledge that the fears expressed with the arguments are based on the literature assessment, in which scholars in the past have pointed towards his possibility and fear scenarios. Furthermore, this policy brief is concerned with the human rights violations that could occur from the ASCN, rather than providing a general overview over the pros and cons of the initiative. Delving into the benefits, therefore, is outside the scope of this article. Neverthleless, I acknowledge this in the second paragraph of section 2: “This policy brief acknowledges that smart city development is geared toward economic progress despite the civic and social elements emphasized in the ASCN.” The revised paragraph: “This section argues that to complement the deficiencies of the ASCN’s smart city conception, two recommendations can be adopted. The first recommendation is to acknowledge the vulnerability of the underprivileged, provide personal security, and promote social inclusion for governing smart cities. As seen in Table 1 below, a prominent problem with Southeast Asian states is the lack of democracy within their social and political landscapes. Consequently, when the ASCN echoed the importance of smart cities, there were fears that this would further derail democratic progress in the region. Studies in the past have shown how smart-city developments risk more substantial possibilities of violating privacy rights as high-tech companies have a stronger voice and role in the planning and financing of smart-city-related projects. ^13–^ ^16^ Half of the Southeast Asian states are authoritarian or semi-authoritarian states. Consequently, there is an intense fear of abuse of power and increased surveillance capacities that are feared would be the consequences of smart city conceptions, albeit, the presence of institutional designs that help to counter the intensity. ^7,^ ^17^”

**Reviewer 2, comment 2.3.2** : Incomplete assessment of local governments’ active role ASCN is described as a top-down imposed network, but in reality: many ASCN projects are proposed by city governments; the level of technology adoption varies greatly across cities. Treating ASCN as a homogeneous structure reduces the accuracy of the argument.

**Response**: I have revised the words used in paragraph 6, section 1. The ‘top-down’ terminology used in the article does not reflect the points used for this policy brief. Rather, it is a quotation of how de Jong in 2023 described the ASCN, which reflects a hierarchical system that does not allow ASEAN member states to adapt towards a regionalized system. The revised paragraph: “A second concern identified in this policy brief is the imposing development models by external actors. The ASCN collaborates and links ASCN members’ projects with possible financing from vast stakeholders. The problem with this is that the ASCN eventually aims to achieve accelerated growth for profit, which includes broader privatization processes along the way. Observing the case of Phnom Penh’s forced privatized lands that disadvantaged people with low incomes, ^11^ a “top-down” (terminology introduced by de Jong in 2023) approach thus risks the livelihood of ASEAN citizens. ^8^ ”

**Reviewer 2, comment 2.3.3** : Critique of China and Japan’s role is overly politicized The paper warns of major powers (BRI, JICA, Huawei) imposing development models. However: it does not compare loan conditions or policy constraints among partners; it does not analyze ASEAN countries’ capacity to negotiate and choose strategies. This makes the argument appear more like geopolitical suspicion than evidence-based development assessment.

**Response**: The reference towards China and Japan’s role here is to simply acknowledge that the systems imposed differs to that of ASEAN member states. Therefore, there is a risk of these imposed systems negatively affecting ASCN mechanisms, which has been the case with Japan and China’s development models argued in several studies in the past (see the citations). Nevertheless, I have revised the paragraph to explain that ASEAN member states do have the capacity to negotiate conditions, and therefore, has the ability to partially counter such concerns. The revised paragraph (Paragraph 6, section 1): “The problem identified with the ASCN’s current model is the acceleration of development under the financing stakeholder’s model. In the past, many of ASEAN’s external partners have partnered with the ASCN pilot cities. ^40 ^Japan has been an active nation, aiming to adopt Japanese models of sustainability and smart city infrastructure through its Japan International Cooperation Agency (JICA), which has increased its presence in Siem Reap, Phnom Penh, and Yangon City. ^10,^ ^11^ China’s Belt Road Initiative (BRI) has also financed many of Southeast Asia’s regional strategic infrastructures. ^41 ^Some have been under the framework of the BRI, while others have been through partnerships with China’s private and public industries. Huawei, for example, was reported to contribute to Thailand’s digital economy and technological updates. ^8,^ ^42^ The problem with this model is that the intention of constructing this smart city leads Southeast Asian states to be affected by the national interests of the foreign states, aiming to impose their development model on the ASCN members. ^43,^ ^44^ Consequently, it will be difficult for recipient cities of the financing to cater to the needs of their vulnerable groups, as attention would be diverted to accelerating the progress of the development in alignment with the interests of the financing stakeholder. Nevertheless, this problem is somewhat countered by the ASEAN member state’s capacity negotiate conditions, which could partially counter these concerns.”

**Reviewer 2, comment 2.4.1:** Human rights recommendation: well-intentioned but impractical Calling for human rights integration into smart city governance is reasonable. However: the paper does not specify concrete institutional mechanisms; it does not clarify who monitors, who implements, and who is accountable. In ASEAN – where no enforcement mechanism exists – this recommendation is more symbolic than substantive.

**Response**: I agree that ASEAN does not have any enforcement mechanisms. Therefore, rather than specifying the details on how this recommendation can be made, I opt to make a slight ‘vagueness’ and choose to simply reference the ASCN as the relevant mechanism that is able to clarify and acknowledge vulnerabilities related to human rights in the context of smart cities. This would allow enough room for ASEAN to determine the best way of mentioning this, without risking violating the ASEAN Way. The explanation in the manuscript is slightly revised in paragraph 2, section 2: “Considering the vulnerability of the underprivileged in larger cities of Southeast Asia, it is essential to consider how past studies have concluded the risks of smart city developments to leave the poor and unemployed in isolation. ^19,^ ^20^ This policy brief acknowledges that smart city development is geared toward economic progress despite the civic and social elements emphasized in the ASCN. The problem with this is the strong possibility of social inclusion and participation of the people in governance being further hampered, as shown in the conclusions of past studies on this subject. ^21–^ ^25^ Therefore, this destination to a ‘smart city’ status must be made carefully by the ASCN to ensure that, in the process, local governments do not impede upon the fundamental human rights of its citizens in the name of development.”

**Reviewer 2, comment 2.4.2** : ASEAN intra-regional support recommendation: idealized view of Singapore’s role The author suggests Singapore and high-HDI countries support others. However: ASEAN is not the EU, it has no common budget; Singapore has no legal obligation to support other countries; national interests may conflict with the role of “regional donor.”

 This recommendation lacks assessment of political and economic incentives.

**Response**: I agree with this. Therefore, as the last paragraph of section 2 explains: “Therefore, the question would be whether Singapore would be openly willing to assist all of its ASEAN counterparts in establishing a balance between smart city status and sustainable development.” This shows that this policy brief acknowledges that Singapore has the strongest capacity to provide assistances, however, there is no mechanism within ASEAN that is able to enforce this to Singapore. Ultimately, the decision to provide the assistances would solely lie on the hands of the Singapore Government, and is outside the scope of this study to argue how best to approach this.

# Reviewer report: Reviewer response for version 1

**DOI:** 10.5256/f1000research.184179.r432949

**Published:** 2025-12-29

**Authors:** Teija Vainio

**Affiliations:** 1 Tampere University, Kanslerinrinne, Tampere, Finland

The policy brief analyses smart city development in Southeast Asian cities and proposes three policy recommendations based on secondary data from the ASEAN Smart Cities Network.

 Overall, the submission demonstrates a clear structure, meets the criteria for a Policy Brief for F1000Research, and contributes to the discourse on smart city development in Southeast Asian cities.

 The primary recommendation concerns how sustainability is described. While the abstract emphasises human rights and public service delivery, subsequent sections address sustainability objectives and models at a general level. The author should clarify and consistently define the main focus throughout the submission, specifying whether it centres on sustainability aspects such as environmental or economic sustainability, or on human rights and public service delivery within smart city development. If social sustainability is the intended focus, it should be explicitly defined.

Does the paper provide a comprehensive overview of the policy and the context of its implementation in a way which is accessible to a general reader?

Yes

Is the discussion on the implications clearly and accurately presented and does it cite the current literature?

Partly

Are the recommendations made clear, balanced, and justified on the basis of the presented arguments?

Partly

Reviewer Expertise:

smart city development

I confirm that I have read this submission and believe that I have an appropriate level of expertise to confirm that it is of an acceptable scientific standard, however I have significant reservations, as outlined above.

# Response

**Published:** 2026-01-07

**Authors:** bama andika putra

**Affiliations:** University of Bristol School of Sociology Politics and International Studies, Bristol, England, UK

Reviewer 1: The primary recommendation concerns how sustainability is described. While the abstract emphasises human rights and public service delivery, subsequent sections address sustainability objectives and models at a general level. The author should clarify and consistently define the main focus throughout the submission, specifying whether it centres on sustainability aspects such as environmental or economic sustainability, or on human rights and public service delivery within smart city development. If social sustainability is the intended focus, it should be explicitly defined

 Response: I agree that the terminology of sustainability needed to be clarified in the introduction section. In the revised paragraph (paragraph 1, section 1), I clarified that elements of sustainability in this case is civic and social. Meanwhile, the third paragraph in section 1 clarifies that civic and social in this case includes “…social cohesion, culture and heritage, tourism, public and municipal services, and governance.” The revised paragraph: “The ASCN is the Association of Southeast Asian Nations (ASEAN) solution towards urbanization and city-based challenges. In 2018, Singapore led the initiative to establish the ASCN, perceiving that a ‘smart city’ conception of Southeast Asian states would assist Southeast Asian nations in countering urbanization-perpetuated challenges and the lack of development in larger ASEAN-located cities. ^1 ^As Ludher argues, “Most of ASEAN’s growth has been and will continue to be driven by urban centers, with more people expected to urbanize by 2030”. ^2 ^This policy brief perceives that throughout the process, the ASCN lacks proper consideration towards human rights and elements of sustainability (civic and social dimensions) in its plan to accelerate the growth of its member cities.”
